# A study of text classification algorithms for live-streaming e-commerce comments based on improved BERT model

**DOI:** 10.1371/journal.pone.0316550

**Published:** 2025-04-22

**Authors:** Rong Zhou, Qing Shen, Huafeng Kong

**Affiliations:** 1 Faculty of Business and Economics, University of Malaya, Kuala Lumpur, Malaysia; 2 Department of Information Engineering, Wuhan Business University, Wuhan, China; Xi'an Jiaotong University, CHINA

## Abstract

As e-commerce live streaming becomes increasingly popular, the textual analysis of bullet comments is becoming more and more important. Bullet comments is characterized by its brevity, diverse content, and vast quantity. Faced with these challenges, this study proposes an improved BERT model based on a hierarchical structure for classifying e-commerce bullet comments. First, a parent class BERT model is trained to categorize bullet comments into six designated categories (parent categories). Subsequently, subclass BERT models are trained to classify bullet comments into subcategories. The model combines BERT’s profound semantic comprehension with the closely categorized capabilities of the hierarchical structure. Empirical evidence shows that the proposed model significantly improves classification accuracy and efficiency, aiding in further analysis of bullet comments, extracting valuable information, and achieving effective marketing.

## 1. Introduction

The digital revolution has catalyzed a surge in the popularity of e-commerce live streaming, which has quickly become a focal point of global attention and a new frontier in online shopping [1]. This innovative approach to retail has been particularly notable in China, where the live streaming e-commerce industry has experienced explosive growth [2]. By 2022, the live streaming e-commerce industry in China had reached a new peak, with a transaction volume of 3,487.9 billion yuan [3]. Live streaming e-commerce enables viewers to send and display real-time comments on the live video stream [4]. These comments, known as ‘bullet comments’ or ‘danmu’, appear on the video in a scrolling, floating, or stationary manner, offering a novel way for viewers to engage with the live broadcast [5]. Viewers can post comments and feedback in real-time during the live stream, allowing hosts to quickly respond to audience needs and questions by monitoring these bullet comments [6]. Furthermore, other viewers can glean product information and purchasing experiences from these comments, which can inform their own buying decisions [7]. The application of bullet comments in live streaming e-commerce not only enriches the presentation of live broadcasts but also introduces new interactive and marketing opportunities for the e-commerce industry. Consequently, it is essential to perform text classification on these comments to gain a deeper understanding of viewer preferences, thereby enhancing marketing efficiency.

Within the realm of text classification techniques, the prevailing approaches encompass classification utilizing semantic dictionaries, conventional machine learning methodologies, and deep learning strategies. Meng, Duan [1] employed a sentiment dictionary to analyze viewer comments in live-streaming e-commerce, a method that, while simple to implement, has inherent limitations in accuracy. Dragoni, Federici [8] explored the application of traditional machine learning for comment categorization; however, this technique faces challenges when applied to extensive datasets. The BERT model from deep learning has garnered widespread recognition for its superior performance in addressing such challenges [9, 10].

Currently, a significant portion of scholarly research is conducted using flat text classification techniques, as evidenced by the works of Cao, Sun [11] and Su, Cheng [12]. These methods involve directly categorizing text data into a predefined set of non-hierarchical categories, without considering the potential for nested or hierarchical relationships among these categories [11, 12]. Despite various enhancements made, the accuracy of these flat classifications has not seen substantial improvement. In contrast, Ma, Liu [13] introduced a hierarchical classification approach and, through a series of five experiments, demonstrated that this method surpasses flat classification in precision. Hierarchical classification organizes categories into a structured hierarchy, where each category functions as a node within a tree-like architecture [14]. This structure allows for categories to have subcategories (child nodes) and a superior category (parent node), creating a directed acyclic graph (DAG) that facilitates a more nuanced and systematic approach to classification [15, 16]. Building upon these insights, this study proposes a hierarchical classification method to augment the BERT model. By incorporating a hierarchical structure into the classification process, we anticipate a more accurate text classification that can better capture the complexities and nuances of viewer feedbacks within the live-streaming e-commerce ecosystem.

Therefore, this study introduces an enhanced BERT model structured hierarchically for classifying e-commerce live broadcast bullet comments. Initially, a parent class BERT model is trained to categorize bullet comments into six predefined categories (parent categories). Following this, the subclass BERT models are trained to further classify the bullet comments into subcategories. This model leverages BERT’s deep semantic understanding, complemented by the hierarchical structure’s ability to tightly classify closely related categories. Empirical evidence demonstrates that the proposed model notably enhances classification accuracy and efficiency.

The contribution of this study is the construction of an innovative BERT model, based on a hierarchical structure. Through this new BERT model, researchers can extract valuable information from bullet comments with the characteristics of brevity, diverse content and vast quantity, which is important for understanding and interpreting viewers’ preferences.

The remaining parts of this paper are organized as follows: Section 2 Related Work; Section 3 Method; Section 4 Experiments; Section 5 Results and Discussions and Section 6 Conclusions and Future work.

## 2. Related work

### 2.1 Text classification

The text classification process, a pivotal domain within Natural Language Processing (NLP), is utilized for a variety of applications such as Sentiment Analysis, Topic Labeling, Question Answering, Dialog Act Classification, and Natural Language Inference [17]. This methodology involves several key steps: obtaining original data, data preprocessing, feature extraction, classifier application, and the generation of category outputs [18]. Original data is often sourced from platforms like Facebook, Twitter, and e-commerce websites, using Python for extraction [19]. Data preprocessing is a crucial step that includes data cleaning to remove noise, standardizing text categories (e.g., Chinese or English), employing word segmentation techniques, and filtering out stop words [20]. Feature extraction can be approached in various ways; traditional methods like the N-gram and TF-IDF are common, while deep learning often relies on automatic feature extraction. Classifiers such as SVM and SoftMax are employed to determine the text’s final characteristics [8]. Text classification techniques encompass semantic dictionary-based methods, traditional machine learning, and deep learning strategies.

Semantic dictionary-based classification uses a specialized dictionary for text identification and categorization. This method involves text input, preprocessing, segmentation, training with the semantic dictionary, and classification based on established rules [9]. For example, Meng, Duan [1] used an emotional dictionary to analyze viewer comments on live streams, exploring emotional contagion. However, this approach, while simple, has limitations in accuracy [9].

Machine learning approaches involve training models on text classification tasks, extracting features from extensive text corpora, and predicting classifications. These methods are categorized into supervised, semi-supervised, and unsupervised learning [8]. Dragoni, Federici [8] employed an unsupervised method for classifying real-time comments. Despite their higher accuracy, machine learning methods may not be ideal for very large datasets [9].

Deep learning methods encompass a range of techniques from single neural networks to hybrid models, attention mechanisms, and pre-trained models [18]. Pre-trained models, like Google’s BERT introduced in 2018, are a focal point of current research. They offer the advantage of capturing intricate lexical relationships and can be fine-tuned for excellent performance in specific tasks [21]. BERT uses a bidirectional mechanism for contextual word understanding and combines Word Piece embeddings with positional encoding [22]. Araci [23] achieved notable success with BERT for text classification due to its high accuracy and suitability for large datasets [9]. In the past few years, the BERT model has gained popularity for its effectiveness in classifying review text. For example, Su, Cheng [12] used an improved BERT model to classify social media comments, which helped in tracking the shifts in public sentiment. Similarly, Cao, Sun [11] applied an enhanced BERT model for sentiment analysis on reviews of agricultural products.

### 2.2 Hierarchical structure

The flat text classification method treats text data in a straightforward manner, ignoring any hierarchical structures [14]. Despite efforts to improve this method, there has not been a significant leap in classification accuracy. Several factors contribute to this challenge. Text classification models are highly complex with numerous parameters, indicating their proficiency in capturing linguistic nuances, which means that further modifications may yield only marginal performance improvements [15]. Additionally, the quality and size of the training data can limit the model’s ability to generalize and enhance accuracy if the dataset lacks diversity or is not large enough [20]. Lastly, the complexity inherent in certain text classification tasks can be problematic [22]. This complexity may stem from the subjective nature of the text, ambiguities within it, or the need for domain-specific knowledge that may not be captured by models pre-trained on general text [24].

Ma, Liu [13] highlighted that traditional flat classification methods struggle with the vast volume and subtle distinctions between categories in real-world text classification scenarios. To address this, they proposed a hierarchical classification method that is more adept at managing complex text classification challenges. Their experiments across five real-world datasets showed that hierarchical classifiers generally outperformed flat classifiers. Hierarchical classification methods offer a structured approach to text classification, particularly useful for tasks with large volumes of text and closely related categories [25]. These methods organize classes into a tree-like hierarchy, with nodes representing categories and edges showing the relationships between parent and child nodes, allowing for a more nuanced classification process [26].

Unlike flat classification systems, which assign texts to one or more categories at the same level and can lead to confusion with overlapping categories, hierarchical approaches provide clarity [27]. They establish a nested structure that organizes similar categories, enhancing classification precision [28]. As the number of texts and categories grows, flat models may become inefficient due to the curse of dimensionality [29]. Hierarchical classification, however, breaks down the classification problem into smaller, more manageable tasks, making it more effective for handling a larger number of categories [30]. It also allows for the addition of new categories without requiring a complete retraining of the model, offering flexibility for dynamic classification needs [31]. Furthermore, hierarchical classification improves the interpretability of the classification process by providing a clear sequence of decisions that led to a particular classification [32]. It also excels at distinguishing between closely related or overlapping categories by training the model to differentiate based on hierarchical relationships [33].

Considering the vast amount of bullet comments in live streaming e-commerce and the closeness of classification categories, to improve classification accuracy, this study employs a hierarchical classification approach.

## 3. Method

**Fig 1:**
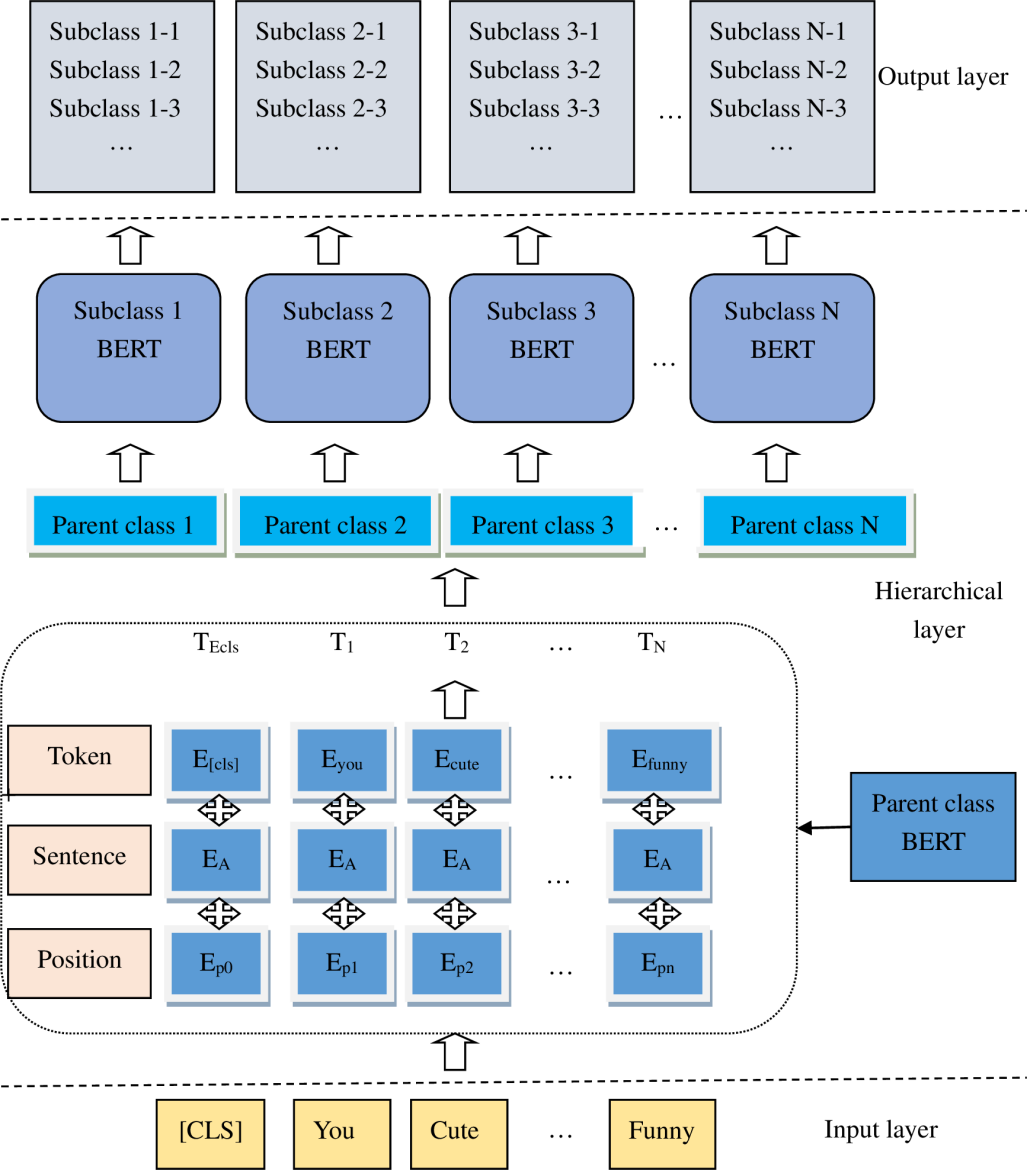
The Structure of the Hierarchical BERT Model.

A BERT model based on hierarchical structure is proposed, referred to as HS-BERT. This is a tiered text classification method where categories are not flat but form a tree-like structure, with some categories being subcategories of others. The parent category is classified into N subcategories using the parent class BERT model, and each subcategory is further classified using a respective subclass BERT model. The framework of our HS-BERT method is illustrated in the Fig 1.

The first is Input Layer. The input layer receives the user’s review sentence s, which is composed of a series of word elements expressed as


$$s=\left\{w{\left(s\right)}_{1},w{\left(s\right)}_{2},\ldots ,w{\left(s\right)}_{n}\right\}$$ (1)

Hierarchical layer is followed. There are two parts. 1) Top-level Classification: First, a BERT model is used to classify the text to determine which top-level parent category it belongs to. This step involves training a parent class BERT classifier that can identify the most likely parent category for the text. 2) Subcategory Classification: Once it is determined which parent category the text belongs to, the next step is to use subclass BERT models to further classify the text into the respective subcategories.

The BERT model is divided into six sections. The first is Word Embeddings. Initially, each word in the sentence is converted into a word embedding vector.


$$E=Embedding\left(words\right)$$ (2)

wordsrefers to the sequence of words in a sentence. *E* refers to the word embedding matrix, where each word is mapped to a fixed-dimensional vector space.

The second is Positional Encoding. Positional encodings are added to the word embeddings to provide information about the position of the words in the sentence.


X=E+P (3)

*P*represents the positional encoding, a vector of the same dimension as the word embedding, whose values are generated by sine and cosine functions. *X* is the input vector that combines word embeddings and positional information. The specific calculation method for positional encoding:


$$PE\left(pos,2i\right)=sin\left(\frac{pos}{10000\frac{2i}{d_{model}}}\right)$$ (4)

$$PE\left(pos,2i+1\right)=cos\left(\frac{pos}{10000\frac{2i+1}{d_{model}}}\right)$$ (5)

posrefers to the position of the word in the sentence. *i* refers to the index of the dimension. $d_{model}$ refers to the word embedding dimension of the model.

The third is Segment Embeddings. When processing segments, different embeddings are used to distinguish between the two sentences.


$$X_{seg}=Eembedding\left(segments\right)$$ (6)

segmentsindicate the type of each sentence (e.g., the first sentence or the second sentence). $X_{seg}$ is the type embedding for the segment.

The fourth is Muti-head Self-Attention. In each encoder layer, the self-attention mechanism allows the model to focus on multiple positions simultaneously when processing a sentence.


$$Q,K,V=XW^{Q},XW^{K},XW^{V}$$ (7)

$$Attention\left(Q,K,V\right)=Softmax\left(\frac{QK^{T}}{\sqrt{d_{k}}}\right)V$$ (8)

Q,K,Vrepresent the query, key, and value matrices. $W^{Q},W^{K},W^{V}$ are learnable weight matrixes. $d_{k}$ is the dimension of the key vectors.

The fifth is Residual Connection and Layer Normalization. The output of each sub-layer (self-attention or feed-forward network) goes through a residual connection and layer normalization.


$$L=LN\left(A+X\right)$$ (9)

*L*is the output after layer normalization and residual connection. LN represents the layer normalization operation.

The last is Feed-Forward Neural Network. Each encoder layer also includes a feed-forward network for further processing the output of the self-attention layer.


$$F=\max\left(0,LW_{1}+b_{1}\right)W_{2}+b_{2}$$ (10)

$W_{1},W_{2},b_{1},b_{2}$represent the learnable weights and biases of the feed-forward network.

The final layer is the output layer.

## 4. Experiments

### 4.1 Data acquisition and preprocessing

In this research, viewer comments on TikTok’s Chinese version have been gathered. The decision to focus on China as the research subject is based on the swift growth of live-streaming e-commerce in the nation since 2016, along with a persistent rise in market penetration [1]. The platform of TikTok (Chinese version), which boasts 700 million daily active users in China, is selected for the study due to its extensive reach among the country’s populace of 1.4 billion [34].

Two data sets are selected in Table 1. Tea and Snacks datasets focus on bullet comments from tea and snacks sales from January 1, 2023, to April 18, 2023 and is publicly accessed on the Chanmama Data Platform (https://www.chanmama.com/). Data collection follows the Chanmama Data Platform’s Service Agreement (https://www.chanmama.com/other/serviceAgreement.html) and Privacy Policy (https://www.chanmama.com/other/privacyAgreement.html).

**Table 1:** Two datasets.

Dataset Name	Sample
Tea	16,224
Snack	12,979

This paper annotates viewer comments. The parent category annotation divides the reviews into 6 categories based on the work of Shen, han Wen [3], namely evaluation, inquiry, promotion, price, logistics, and influencer, followed by manual annotation. The subcategory annotation further classifies the parent categories based on existing literature. Specifically, the evaluation category is broken down into three subcategories: quality, packaging, and after-sales service [1]. The inquiry section is divided into six parts: variety, quantity, quality, origin, packaging, and price [35]. The promotion category is subdivided into three subcategories: scarcity promotions, monetary promotions, and non-monetary promotions [36]. The price category is split into two subcategories: expensive and cheap [1]. The logistics category is detailed into six subcategories: reliability, economy, empathy, timeliness, flexibility, and informativity [37]. Lastly, the influencer category is divided into four subcategories: professionalism [38], homogeneity [2], attraction [39], and interactivity [40]. Manual annotation is followed. The corresponding labels can be found in Table 2 and 3. The data is randomly sorted with 80% as the training set and 20% as the validation set.

**Table 2:** Parent class classification label.

Parent class	Description
Evaluation	The text contains evaluations of product performance.
Inquiry	The text contains inquiries about product information.
Promotion	The text includes reactions to promotional strategies.
Price	The text includes feedback on product price.
Logistics	The text includes feedback on shipping services.
Influencer	The text includes reactions to live streaming influencers.

**Table 3:** Subclass classification label.

Parent class	Subclass	Description
Evaluation	Quality	The text contains evaluations of the quality of the product, including durability, performance, materials and so on.
	Packaging	The text contains evaluations of the product packaging, concerning the aesthetics of the packaging, protection, etc
	After-sales	The text contains comments on after-sales service, such as return and exchange policies, customer service response speed and service quality
Inquiry	Variety	The text contains queries about product varieties
	Quantity	The text contains queries about the number of products
	Quality	The text contains inquiries about the quality of the product
	Origin	The text contains queries about the origin of the product
	Packaging	The text contains queries about product packaging
	Price	The text contains inquiries about the price of the product
Promotion	Scarcity promotions	The text contains responses to scarcity promotions such as limited-quantity, limited-time, etc
	Monetary promotions	The text contains responses to monetary incentives such as price concessions, discounts, and buy-one-get-one-free offers.
	Non-monetary promotions	The text contains responses to non-monetary incentives such as gifts, points, coupons, etc
Price	Expensive	The text contains feedback on high price
	Cheap	The text contains feedback on low price
Logistics	Reliability	The text contains evaluations of the reliability and safety of logistics services
	Economy	The text contains evaluations of logistics costs
	Empathy	The text contains the evaluation of customer service in the process of logistics service
	Timeliness	The text contains comments on the speed of logistics, including delivery and distribution
	Flexibility	The text includes evaluations of the flexibility of logistics services, such as the ability to specify delivery time or locations
	Informativity	The text contains evaluations of the transparency and frequency of updates regarding logistics information
Influencer	Professionalism	The text contains feedback on the influencer’s expertise.
	Homogeneity	The text contains feedback on the influencer’s homophily
	Attraction	The text contains feedback on the influencer’s attractiveness
	Interactivity	The text contains feedback on the influencer’s interactivity

### 4.2 Experimental environment

The experimental setup utilized computing hardware in the form of an NVIDIA GeForce RTX 3090 with 64GB of memory, and the deep learning framework employed was PyTorch [41]. The detailed specifications of the experimental environment are depicted in Table 4.

**Table 4:** Experimental environment.

Projects	Configuration
Operating Platforms	CUDA 10.1
Operating System	Windows 11
Memory	64GB
Python Versions	Python 3.8.1
Pytorch Versions	Pytorch 2.2

### 4.3 Model parameter setting

The relevant parameters of the model are shown in Table 5 based on the work of Shen, han Wen [3]. The selection of an optimizer is pivotal as it can markedly influence the outcomes of model training [42]. Guan [43] examined the performance of Adam and AdamW optimizers. The findings indicated that AdamW, when integrated with weight decay (denoted as λ), demonstrated superior efficacy in regularizing the model and curbing overfitting tendencies. Analyzing the impact of learning rates on the loss curve is essential for finding the optimal rate, ensuring model stability, and speeding up convergence. Monitoring epochs’ effect on accuracy helps in tracking learning progress, detecting overfitting, and enhancing the model’s generalization. Adjusting dropout rates may necessitate more epochs for learning, while adding transformer layers should be paired with an increase in hidden neurons to maintain representational power. Varying convolution sizes and kernel sizes allows the model to capture features at different scales.

**Table 5:** Model parameter settings.

Hyper-parameters	Value
Learning rate	1e-5
Loss Function	Cross-Entropy Loss
Optimizer	AdamW
Batch size	32
Dropout	0.1
Convolution size	3×3
Kernel sizes	[3–5]
Epochs	10
Transformer layers	12
Transformer number of hidden neurons	768

To assess the effectiveness of the models, performance metrics including accuracy, precision, recall, and the F1 score are applied. In the context of classification, instances where positive instances are correctly identified are termed True Positives (TP), while those incorrectly classified as negative are known as False Negatives (FN). Conversely, cases where negative instances are incorrectly labeled as positive are referred to as False Positives (FP), and when negative instances are correctly identified, they are called True Negatives (TN).


$$Accuracy=\frac{TP+TN}{TP+FP+TN+FN}$$ (11)

$$Precision=\frac{TP}{TP+FP}$$ (12)

$$Recall=\frac{TP}{TP+FN}$$ (13)

$$F1=\frac{2*Precison*Recall}{Precision+Recall}$$ (14)

## 5. Results and discussions

Since HE-BERT is structured hierarchically, following the work of Ma, Liu [13], we only need to obtain the accuracy, precision, recall, and F1 score of the subclass BERT models, as shown in Table 6. To facilitate comparison with other models, we take the average of these four metrics of the subclass BERT models.

**Table 6:** Four Indexes of HE-BERT’s Subclass Models on Tea and Snacks Dataset.

	Tea				Snacks			
BERT model	Accuracy	Precision	Recall	F1	Accuracy	Precision	Recall	F1
Subclass 1	99.42%	99.49%	99.38%	99.44%	98.91%	98.92%	98.76%	98.84%
Subclass 2	97.29%	80.85%	80.99%	80.91%	96.28%	96.30%	96.05%	96.16%
Subclass 3	97.63%	97.67%	97.64%	97.65%	96.90%	96.89%	96.89%	96.89%
Subclass 4	99.43%	99.54%	99.26%	99.40%	99.37%	99.34%	99.34%	99.34%
Subclass 5	98.17%	97.28%	98.02%	97.63%	93.36%	92.69%	91.90%	92.15%
Subclass 6	97.39%	97.60%	97.56%	97.54%	94.62%	63.01%	63.46%	63.19%
Average	98.22%	95.41%	95.48%	95.43%	96.57%	91.19%	91.07%	91.10%

**Fig 2:**
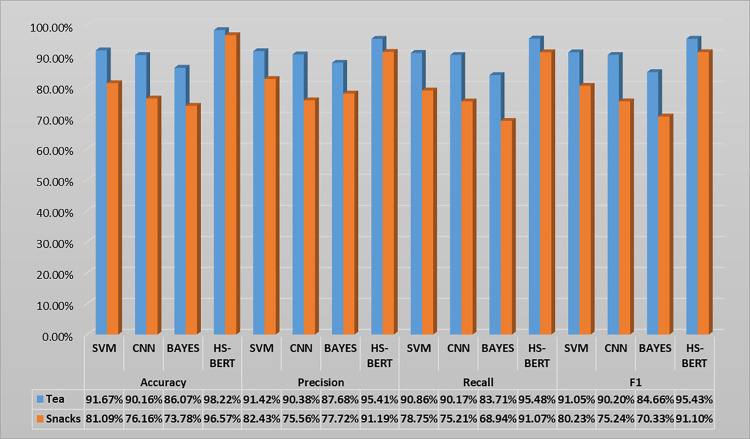
Comparison of HS-BERT and Other Text Classification Methods.

The efficacy of the HS-BERT model is compared against baseline research. The model is evaluated in comparison with SVM, CNN, and BAYES models. Evaluation metrics include accuracy, precision, recall and F1 score. Comparison results are in Fig 2.

On the Tea dataset, the accuracy for SVM, CNN, BAYES, and HS-BERT are 91.67%, 90.16%, 86.07%, and 98.22%, respectively. Notably, HS-BERT outperformed the other models in terms of accuracy. In terms of precision, the respective rates are 91.42% for SVM, 90.38% for CNN, 87.68% for BAYES, and 95.41% for HS-BERT, with HS-BERT again showing superior performance. For recall, the figures are 90.86% for SVM, 90.17% for CNN, 83.71% for BAYES, and 95.48% for HS-BERT, where HS-BERT demonstrated higher recall than the other models. Lastly, considering the F1 score, the results are 91.05% for SVM, 90.20% for CNN, 84.66% for BAYES, and 95.43% for HS-BERT. In all four evaluation metrics—accuracy, precision, recall, and F1 score—HS-BERT surpassed the other models.

On the Snack dataset, the accuracy for SVM, CNN, BAYES, and HS-BERT are 81.09%, 76.16%, 73.78%, and 96.57%, respectively. HS-BERT demonstrates the highest accuracy among the models. When it comes to precision, the rates are 82.43% for SVM, 75.56% for CNN, 77.72% for BAYES, and 91.19% for HS-BERT, with HS-BERT leading in precision. For recall, the figures are 78.75% for SVM, 75.21% for CNN, 68.94% for BAYES, and 91.07% for HS-BERT, where HS-BERT shows the highest recall. In terms of the F1 score, the results are 80.23% for SVM, 75.24% for CNN, 70.33% for BAYES, and 91.10% for HS-BERT. Across all four metrics—accuracy, precision, recall, and F1 score—HS-BERT consistently outperforms the other models.

Experiments conducted on two distinct datasets have demonstrated that HS-BERT outperforms traditional text classification methods in terms of performance for live streaming e-commerce bullet comments. The role of live streaming bullet comment classification is pivotal in enhancing the efficiency of live streaming marketing, with its benefits highlighted in several key areas: 1) Information Extraction. It extracts crucial insights from the bullet screens, such as audience preferences, inquiries, or suggestions about the live content, providing real-time feedback to the anchor. 2) Trend Analysis. By categorizing the bullet screens, anchors can identify trending topics or patterns within the live stream, guiding content creation. 3) Audience Interaction. Classification of bullet screens helps identify audience feedback, enabling anchors to engage more effectively with viewers. These functionalities contribute significantly to the enhancement of live streaming marketing effectiveness.

## 6. Conclusions and future work

This research introduces an advanced BERT model, architected with a hierarchical framework, specifically tailored for the classification of e-commerce bullet comments characterized by their conciseness, diverse content, and substantial volume. Initially, the parent class BERT model is deployed to categorize the bullet comments into six predefined parent categories. Following this, the subclass BERT models are trained to further distinguish comments into their respective subcategories. This approach leverages BERT’s sophisticated semantic understanding, synergistically enhancing it with the hierarchical structure’s meticulous categorization capabilities. Empirical data indicates that the model in question markedly enhances the precision and efficiency of classification, facilitating deeper analysis of bullet comments, extracting actionable insights, and enabling targeted marketing strategies within the dynamic landscape of live streaming e-commerce.

To makes HS-BERT model persuasive and general, we plan to broaden it to encompass a range of linguistic contexts, extending beyond English to include French, Spanish, and numerous other languages. However, it is essential to recognize the challenges faced by HS-BERT, especially when considering the differences between alphabetic and character-based languages. For alphabetic languages such as English and Spanish, issues primarily revolve around the complexity of grammar, rich morphology, polysemy, and spelling variations. In the case of character-based languages like Japanese and Korean, challenges include the absence of clear word boundaries, difficulties in word segmentation, smaller corpus sizes, larger character sets, heavy contextual dependence, and a multitude of homophones and homographs. Addressing these language-specific hurdles is crucial for enhancing the model’s effectiveness across diverse linguistic contexts. Furthermore, to enhance the generalization ability of HS-BERT model, we will test it on the datasets of e-commerce product reviews and social media reviews.

Additionally, within the sphere of live-streaming e-commerce, a troubling pattern of counterfeit viewer reviews has emerged. Influencers and businesses often hire ‘professional reviewers’ to post inauthentic positive reviews or inundate live streams with comments to artificially boost a product’s exposure and perceived favorability. This tactic fosters a false impression of superior product quality and high sales, deceiving potential buyers. These reviewers coordinate a significant number of individuals to carry out fraudulent order placements and provide interactive engagement during live streams, all in service of creating a deceptively positive image for the brand. Additionally, they utilize cloud control systems that enable a single phone to control multiple devices simultaneously for posting reviews, further manipulating the data to their advantage. The proliferation of inauthentic comments poses a significant threat to the understanding of viewer preference. As a result, the development of robust methods to detect and eradicate these deceptive reviews has become a pressing issue.

## Supporting information

S1 Data Supporting information data. (ZIP)
